# Ice accommodation in plant tissues pinpointed by cryo-microscopy in reflected-polarised-light

**DOI:** 10.1186/s13007-020-00617-1

**Published:** 2020-05-20

**Authors:** Matthias Stegner, Johanna Wagner, Gilbert Neuner

**Affiliations:** Department of Botany, Unit Functional Plant Biology, University of Innsbruck, Sternwartestrasse 15, 6020 Innsbruck, Austria

**Keywords:** Birefringence, Ice crystal, Ice localisation, Ice segregation, Ice management, Freezing resistance

## Abstract

**Background:**

Freezing resistant plant organs are capable to manage ice formation, ice propagation, and ice accommodation down to variable temperature limits without damage. Insights in ice management strategies are essential for the fundamental understanding of plant freezing and frost survival. However, knowledge about ice management is scarce. Ice crystal localisation inside plant tissues is challenging and is mainly based on optical appearance of ice in terms of colour and shape, investigated by microscopic methods. Notwithstanding, there are major uncertainties regarding the reliability and accuracy of ice identification and localisation. Surface light reflections, which can originate from water or resin, even at non-freezing temperatures, can have a similar appearance as ice. We applied the principle of birefringence, which is a property of ice but not of liquid water, in reflected-light microscopy to localise ice crystals in frozen plant tissues in an unambiguous manner.

**Results:**

In reflected-light microscopy, water was clearly visible, while ice was more difficult to identify. With the presented polarised cryo-microscopic system, water, including surface light reflections, became invisible, whereas ice crystals showed a bright and shiny appearance. Based on this, we were able to detect loci where ice crystals are accommodated in frozen and viable plant tissues. In *Buxus sempervirens* leaves, large ice needles occupied and expanded the space between the adaxial and abaxial leaf tissues. In *Galanthus nivalis* leaves, air-filled cavities became filled up with ice. Buds of *Picea abies* managed ice in a cavity at the bud basis and between bud scales. By observing the shape and attachment point of the ice crystals, it was possible to identify tissue fractions that segregate intracellular water towards the aggregating ice crystals.

**Conclusion:**

Cryo-microscopy in reflected-polarised-light allowed a robust identification of ice crystals in frozen plant tissue. It distinguishes itself, compared with other methods, by its ease of ice identification, time and cost efficiency and the possibility for high throughput. Profound knowledge about ice management strategies, within the whole range of freezing resistance capacities in the plant kingdom, might be the link to applied science for creating arrangements to avoid future frost damage to crops.

## Background

Low-temperature resistance of plants is highly diverse, ranging from chilling susceptible species in tropical biomes to extreme freezing resistant species of the boreal zones that even survive immersion in liquid nitrogen in the dormant state. Stress caused by low temperature limits global plant distribution, threatens crop survival and by this can lead to tremendous economic damage [1]. A challenging factor for frost survival is ice management, that is where it initially forms, how it propagates, and how it aggregates in certain places and is generally accommodated within the plant body [2]. Investigation of ice management has largely been neglected when compared with other aspects of freezing resistance [2]. Presumably, this is due to difficulties in visualising ice in plant tissues as we lack simple and unambiguous detection techniques. Nevertheless, knowledge of ice accommodation in plant tissues will be essential for the fundamental understanding of frost survival of plants within the whole range of freezing resistance capacities (< 0 °C to − 196 °C). Additionally, this might be the basis for the applied sciences to create improved arrangements for frost protection of crops in the future [2].

For the investigation of ice management processes in plant tissues various methods have been employed [1, 3]: differential thermal analysis (DTA) [4, 5], infrared imaging [6–18] and in particular infrared differential thermal analysis (IDTA), nuclear magnetic resonance (NMR)/magnetic resonance imaging (MRI) [1, 19–23], microscopic observations [17, 20, 24–36], indirect observation by freeze-substitution EM [37], cryo-scanning electron microscopy (cryo-SEM) [32, 36, 38] and X-ray phase contrast imaging [3]. All these approaches differ largely in the obtained resolution, the necessary expenses, time requirements and the gained information: ice is either directly visualised or indirectly detected by measurement of freezing exotherms or assessed by the remaining amount of liquid water. Not all these methods allow control of cooling rates. Rates greater than 3 K/h can result in artificial supercooling [39] which is critical for frost injury.

Precise temperature measurements can detect ice formation, as the phase transition from liquid water to ice releases measurable heat, the so-called freezing exotherm. Based on this principle DTA compares the sample temperature with a reference temperature and allows to identify freezing, but cannot detect the initial site of ice nucleation or the direction of ice propagation [1]. In contrast, IDTA allows visualising the spatiotemporal ice propagation. This enables to detect the point of ice nucleation, the propagation pattern and its speed [6, 7]. As the release of heat is not quantified, both methods only provide a qualitative statement about the intensity of freezing [40]. Although the sensitivity of infrared signal detection improved rapidly, the localisation of ice inside of samples requires invasive sectioning [20].

The supercooling water in plant samples exposed to freezing temperatures can be quantified by NMR spectroscopy [21, 22], while NMR micro-imaging or magnetic resonance imaging (MRI) localises liquid water [1, 19, 20, 23]. Both are noninvasive techniques. However, it remains unclear, whether the loss of proton signal is due to phase transition from solid to liquid or due to desiccation. In contrast to IDTA, MRI visualises the spatio-temporal pattern of unfrozen water, which shows supercooled and non-frozen areas. With MRI, a maximum resolution of 20-100 µm can be achieved, however detection of rapid phenomena at high resolutions is difficult [20, 41]. Application of MRI to study freezing behaviour in florets and buds is established [20, 42], but for leaves there is limited research available [43]. MRI is a powerful tool for investigating freezing behaviour of plants, but there have been only a few high-resolution studies because of the limitation of machine time and expense [20].

Ice crystals can be visualised and located inside of plant tissues by cryo-SEM and/or light microscopy. Microscopic methods require sectioning at a certain point in time; this entails the potential risk of ice crystal formation during incision. Further, temporal monitoring of ice formation in a specimen is not possible [20]. However, microscopy is a standard tool for detection of ice in plant tissues. Light microscopy was mainly applied to identify extracellular ice whereas cryo-SEM in addition can detect intracellular ice [36]. Cryo-SEM is also a valuable tool for monitoring freezing responses of plant tissues or cells [38]. As fixation requires very low temperatures (− 160 °C) [32] a direct observation of naturally formed ice crystals, which have formed prior to the fixation process, is not possible. Only indirect observation of naturally formed ice is possible as it can be distinguished whether ice crystals have formed before or after the fixation process [38]. By observation under vacuum conditions around − 110 °C, there is a risk of ice crystal formation and deformation [20]. In former studies, sectioning was performed by fracturing under vacuum conditions which produced irregular surfaces and structures difficult to interpret [44]. However, techniques to gain flat surfaces were developed by which ice can be detected unambiguously [44]. Nonetheless, a trained eye is crucially important. Overall, the cryo-SEM procedure seems to be elaborate and requires expensive technical equipment.

To directly detect and observe ice in plant tissues, optical light microscopy techniques have been used since the 19th century [30]. Continuous improvements of resolution and particularly image quality gained more detailed insights. Transmitted-light microscopy in temperature-controlled conditions [45–48] and frozen specimens, which are incised and microscopically observed in reflected light [20, 29, 49], are the most common approaches. For transmitted-light microscopy specimens (organisms to cross-sections) are mounted on microscopic slides in liquid water. Indeed, freezing of this water may simulate extracellular ice, which allows monitoring of cellular responses to the presence of ice. Undoubtedly, the amount of frozen water and the location of ice masses inside the tissue are unrealistic and can cause misleading interpretations. To directly localise ice crystals, plant organs can be cut and inspected in reflected-light microscopy. The identification of ice crystals is based on the optical appearance in terms of colour and shape. Unfortunately, this can be a challenging task, as it does not work equally well for all plant tissues. In certain tissues, differentiation between ice crystals, liquid water or resin can be impossible as surface light reflections interfere and cause a similar appearance. Although light microscopic methods are easy and comparably cheap, ambiguousness of ice detection might have triggered the deployment of the much more complex methods presented before.

To improve visibility of ice in plants, a prerequisite is a reliable distinction between the solid and liquid aggregation state of H_2_0. The optical property “birefringence” is a feature of ice but not of liquid water. While water in the liquid state is known to be optically isotropic, ice crystals are optically anisotropic [50]. However, plant tissue constituents can also have birefringence, but if so, they can be detected in unfrozen samples. Birefringence can be assessed by polarised transmitted-light microscopy, which is a standard tool in mineralogy studies for material characterisation in thin sections or powders [51]. The present methodology builds on early research visualising ice with polarised light in frozen *Cornus officinalis* overwintering buds [52], *Lactuca sativa* and *Celastrus orbiculatus* seeds [53].

In this study, we tested whether ice can unambiguously be distinguished from liquid water using polarised light in reflected-light microscopy. To rule out disruptive effects during preparation of thin cross sections and the necessity of a liquid sample carrier for transmitted-light microscopy, we used reflected-light microscopy to inspect frozen plant organs immediately after cutting. Inspections were performed in structural different plant organs from three freezing resistant species: in leaves of an evergreen shrub (*Buxus sempervirens*), in leaves of an herbaceous species (*Galanthus nivalis*) and in overwintering buds of conifers (*Picea abies*).

## Results

### Distinction between water and ice in reflected light

**Fig. 1:**
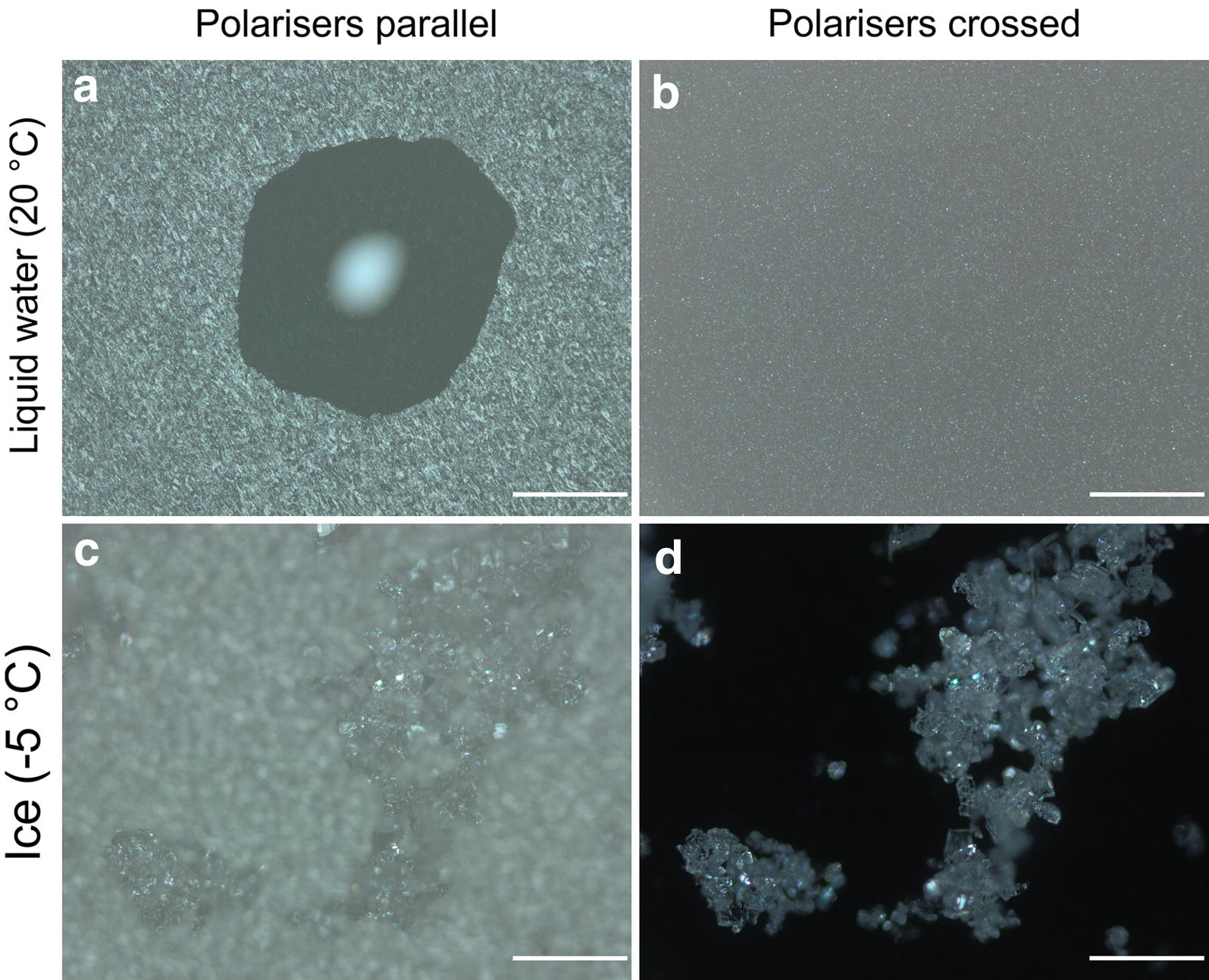
Water in different aggregation states observed with reflected-light microscopy: **a** Image of a droplet of water in polarised light; polarisers are orientated parallel to each other. **b** The same droplet of water disappeared when polarisers are crossed. **c** Ice crystals could hardly be seen with parallel orientated polarisers. **d** Under crossed polarisers, ice crystals became much clearer, while the background completely darkened. (Distance between object and objective was the same in **a**/**b** and **c**/**d**; no image processing was performed). White bars indicate 500 µm

To verify whether liquid water can be reliably distinguished from frozen water, experiments were performed with water in different aggregation states at different temperatures (Fig. 1). By parallel-orientated polarisation filters or without polarisers a droplet of water could clearly be detected in reflected-light microscopy (Fig. 1a). In the centre of the droplet, a strong light reflection occurred. Under crossed polarisers, the droplet of water, including the light reflection, disappeared (Fig. 1b). With parallel polarisers or without polarisers ice crystals were difficult to identify (Fig. 1c). However, under crossed polarisers, ice crystals showed a glowing appearance and could be identified much more explicitly (Fig. 1d).

### Detection of ice in plant tissues

**Fig. 2:**
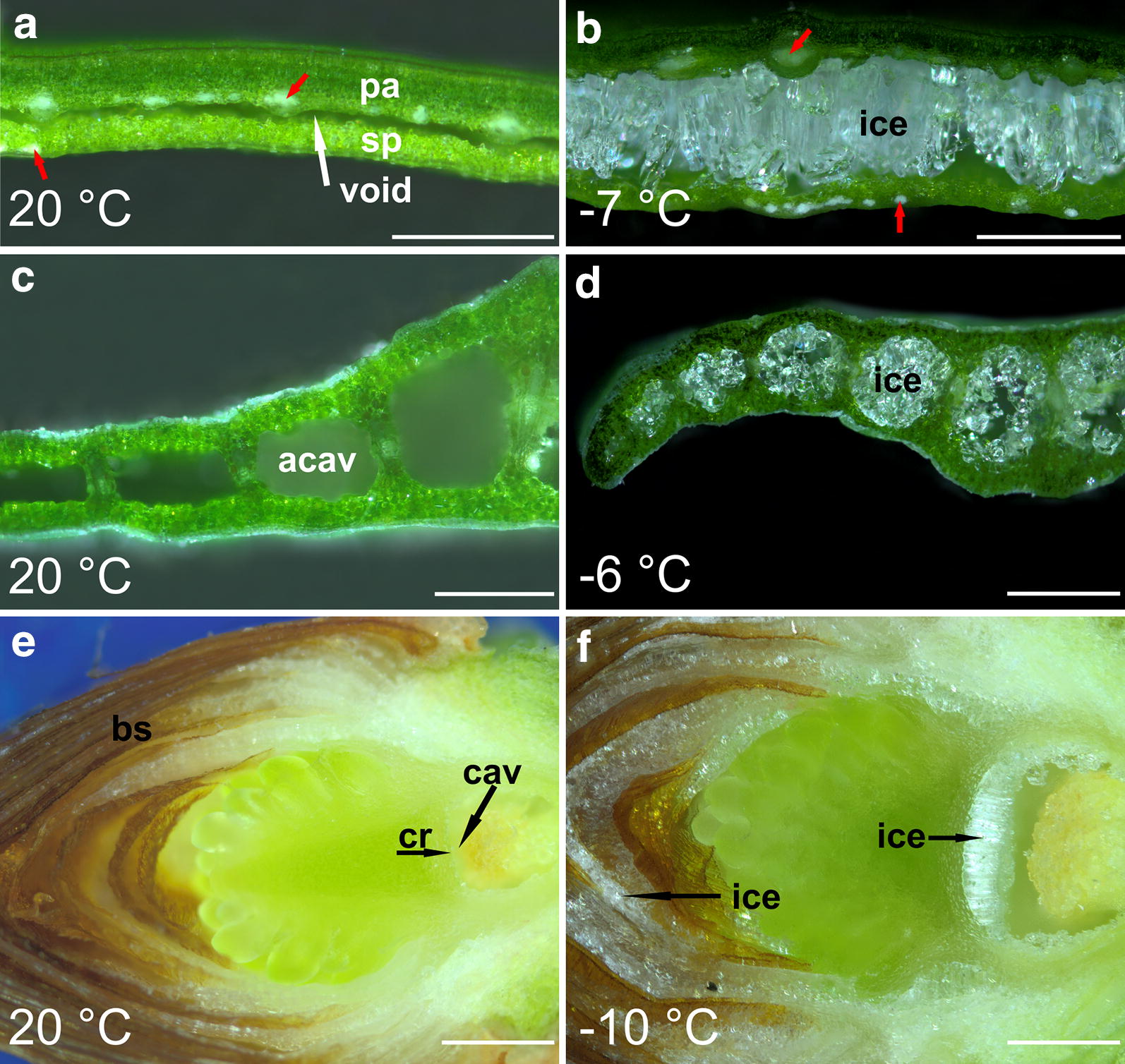
Microscopic observations of different plant organs from various species in reflected-light microscopy: Leaves of *B. sempervirens* (**a**, **b**), leaves of *G. nivalis* (**c**, **d**), and buds of *P. abies* (**e**, **f**) at room temperature and in a frozen state. Entire leaves or shoots with terminal buds were frozen to sublethal target temperatures before sectioning was performed. Ice crystal identification was facilitated by the crossed arrangement of the polarisation filters. In leaves of *B. sempervirens* large ice crystals formed, which expanded the preexistent cavity (void) between the palisade tissue (pa) and spongy tissue (sp), which was formed after the first freeze event. The bright spots (red arrows) are very likely due to other birefringent tissue components, as they are visible at room temperature and in the frozen state (**b**). In leaves of *G. nivalis* ice occurred prominently in the air-filled cavities (acav) (**d**). In buds of *P. abies* ice occurred in the cavity (cav) below the crown (cr) and between the bud scales (bs) during translocated ice formation (**f**). White bars indicate 500 µm

Identification of ice in plant tissues using cryo-microscopy in reflected-polarised-light (CM_rpl_) is demonstrated for three species in Fig. 2:

In unfrozen leaves of *Buxus* *sempervirens* a void between the adaxial leaf side (mainly parenchyma cells and vascular tissue) and the abaxial leaf side (spongy tissue) was found (Fig. 2a). When frozen down to − 7 °C, large ice masses could clearly be identified by their bright shiny appearance in the CM_rpl_-image. Ice masses occupied and expanded the space between the two mesophyll layers, which in the frozen state became completely separated (Fig. 2b). The ice masses consisted of parallel oriented ice needles, originating from the adaxial side, where the vascular tissue is located. Presumably, they aggregated from where cellular water is segregated into the apoplast during freeze dehydration. In the CM_rpl_-image further bright spots in the vascular tissue and in close proximity to the lower epidermis could be discerned (red arrows in Fig. 2a,b). Since these spots appear similar in the unfrozen leaf at + 20 °C, they likely originated from birefringent tissue components other than ice.

Unfrozen leaves of *Galanthus nivalis* showed huge air filled cavities (mean cross sectional diameter = 363 ± 146 (SD) µm) (Fig. 2c). When frozen, ice accumulated in these cavities (Fig. 2d). The ice crystals were irregularly shaped, but attached to the inner surfaces, which produced a hollow-cylindrical or cylindrical appearance.

Buds of *Picea abies* have a complex architecture (Fig. 2e) [24]. The chlorophyll rich new shoot including the needles and the apical meristem are further referred to as “bud”. The bud is separated from the stem by the so-called crown, which forms an air-filled cavity. Multiple layers of bud scales surround the bud. When frozen, in the CM_rpl_-image no ice was found in the supercooled bud, but ice could be clearly identified in various surrounding spaces. Ice formed in the stem below and accumulated in the cavity below the crown and in spaces between the bud scales (Fig. 2f). The compact ice mass formed in the cavity below the crown consists of parallel oriented needle-like columns of ice. These ice masses originated from water segregated across the crown during extra-organ (translocated) ice formation. In the bud scales, bright birefringent structures were detected already in the unfrozen state. Nevertheless, in the CM_rpl_-image at − 10 °C rather irregularly-formed ice crystals between the bud scales could explicitly be identified. However, due to other birefringent components in the scales the extent of these ice masses was difficult to judge.

## Discussion

With the experimental setup we have developed, a robust differentiation between water and ice using polarisation filters in reflected-light microscopy is possible. If light passes through a polariser this yields plane polarised light, which can be extinguished by a second polarisation filter (analyser) when the filters are crossed. However, this only holds if there is isotropic material between the polariser and the analyser. If there is birefringent material in between, this will result in a wave inclined at some angle compared with the original plane wave [50], which will be not fully extinguished by the analyser. By this, birefringent objects become visible under crossed polarisers.

With CM_rpl_, ice could be identified more clearly, whereas liquid water could be made invisible. Water surface reflections can be deceptive in reflected-light microscopy as their distinction from ice is often hardly possible. With CM_rpl_, water surface reflections disappeared under crossed polarisers ruling out potential uncertainties. Similar to water, resins from pines can cause deceptive surface light reflections in reflected-light microscopy. As resins did not show birefringence (unpublished Stegner and Neuner), surface light reflections from resins could also be extinguished by crossed polarisation filters. The only obstacle were birefringent tissue components. However, their potential misleading interpretation can be ruled out by investigation of unfrozen control samples.

### Advantages of CM_rpl_ for detection of ice in plant tissues

By the application of CM_rpl_, it is possible to gain detailed new insights into ice accommodation and segregation strategies in plant tissues. The system allows a rapid inspection of a high number of samples. The acquisition costs are comparably low and operation expenses are negligible. Besides the determination of ice crystals in frozen plant tissues, CM_rpl_ offers additional valuable information about ice aggregation. The resolution is high enough to identify the shape of the apoplastic ice crystals, which can be valid information to understand how they are formed. Additionally, in all three examples (Fig. 2) attachment to surfaces and orientation of ice crystals were clearly visualised. The precise localisation of ice crystals on surfaces indicates from which tissue parts water is segregated towards the ice crystals.

Additional preliminary results showed that lethal intracellular ice formation could be identified in mesophyll cells of *Trachycarpus fortunei*. Hence, with the CM_rpl_, it can be examined where frost damage originates inside plant tissues.

## Conclusion

To understand how plant species with different freezing tolerance limits accommodate ice masses in their organs, without becoming damaged, will be essential for the fundamental understanding of frost survival. Unambiguous ice identification at a high resolution with CM_rpl_ allows profound insights into ice accommodation and aggregation of ice crystals. CM_rpl_ is a valuable tool in addition to the existent pool of methods for assessing ice management in plant tissues. The various methods available provide different information. Hence, combining the results of the various methods will provide the most comprehensive picture for a better understanding of plant freezing processes. The methods differ largely in the time requirement and expense. Also spatial resolution differs greatly (freeze-fracture and freeze-substitution EM > cryo-SEM > light microscopic methods > MRI > IRVT (IDTA) > the unaided eye > DTA [1]). The required level of detail has to be chosen by choosing the appropriate method. CM_rpl_ has major benefits in ease of ice identification, less time requirement, inspection of a high number of samples and cost efficiency.

## Methods

### Plant material

Localisation of ice in plant tissues was studied in leaves of an evergreen shrub (*Buxus sempervirens* L.), in leaves of an herbaceous geophyte (*Galanthus nivalis* L.) and in overwintering buds of a gymnosperm tree (*Picea abies* (L.) H. Karst.). All plants were taken during winter from the field: Leaves of *B. sempervirens* were sampled in December from a single individual in the Botanical Garden of the University of Innsbruck (47°16′4.7′′ N 11°22′47.3′′ E, 606 m a.s.l.), twigs with buds of *P. abies* were sampled in January from seven individuals in the Alpine Garden on Mt. Patscherkofel (47°12′39.2′′ N, 11°27′4.3′′ E, 1919 m a.s.l.), and leaves of *G. nivalis* were sampled in January from two individuals that were cultivated in pots in the Botanical Garden of the University of Innsbruck. All experiments were repeated at least 7 times for each species. For the experiments, samples were either immediately used after collection or, in case of samples from the Alpine Garden, stored for a maximum of 3 days at + 5 °C.

### Freezing protocol and sample preparation

**Fig. 3:**
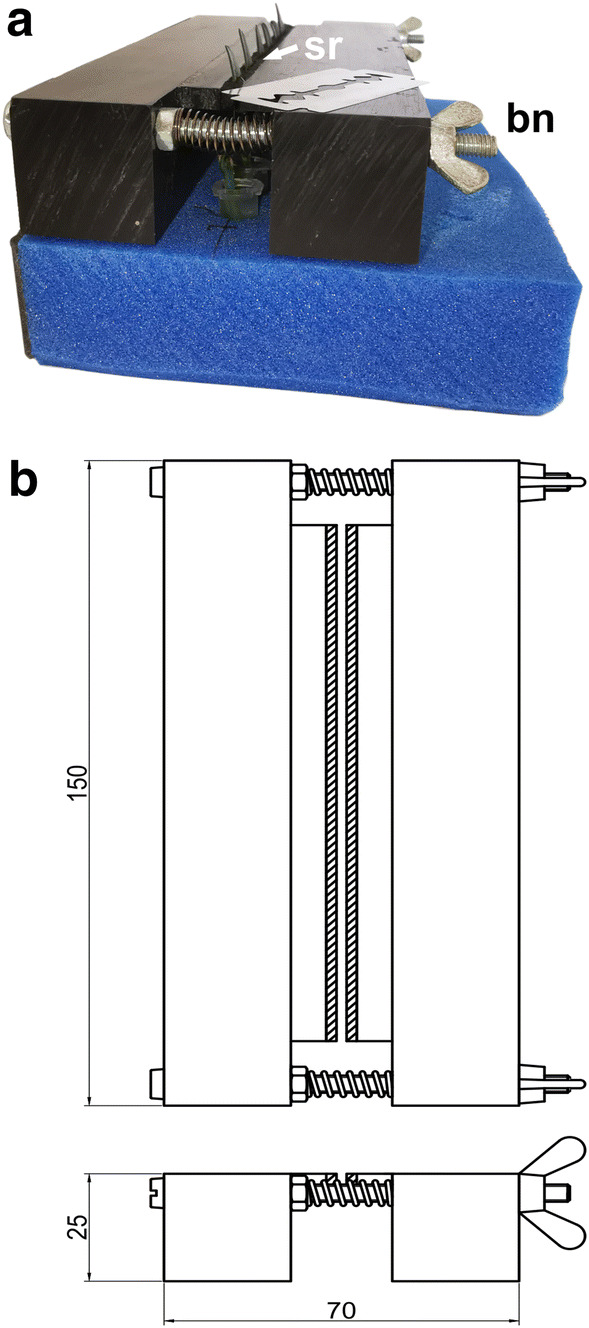
Leaves were clamped between sponge rubber (sr) by tightening the butterfly nuts (bn) (**a**). Leaves were immersed in a solution of water with ice nucleation active (INA) bacteria to trigger ice nucleation and to avoid artificial supercooling. Sectioning was performed with a razorblade, which was guided during cutting by the plane surface of the sample holder. Leaves were cut one at a time and, thereafter, immediately inspected with CM_rpl_. **b** Dimensions in millimetres are shown in the technical drawing; sponge rubber is indicated by the hatched area

For the experiments, entire leaves of *Buxus* (lamina with petiolus) and *Galanthus* (lamina with leaf basis), and about 10 cm long shoots (*Picea*), which were defoliated around the terminal buds, were used. For sectioning under the microscope a special sample holder was designed (Fig. 3): *Buxus* and *Galanthus* leaves were inserted into 0.5 ml Eppendorf tubes filled with distilled water, and leaf blades were clamped between the sponge rubbers of the holder (Fig. 3a). *Picea* shoots with buds were horizontally placed on the top of the sample holder.

For investigation of the unfrozen controls (20 °C), leaves were cross sectioned with a razorblade guided by the sample holder surface. *Picea* shoots were shortened to a length of about 1 cm and buds with the remaining shoot were cut into halves.

**Fig. 4:**
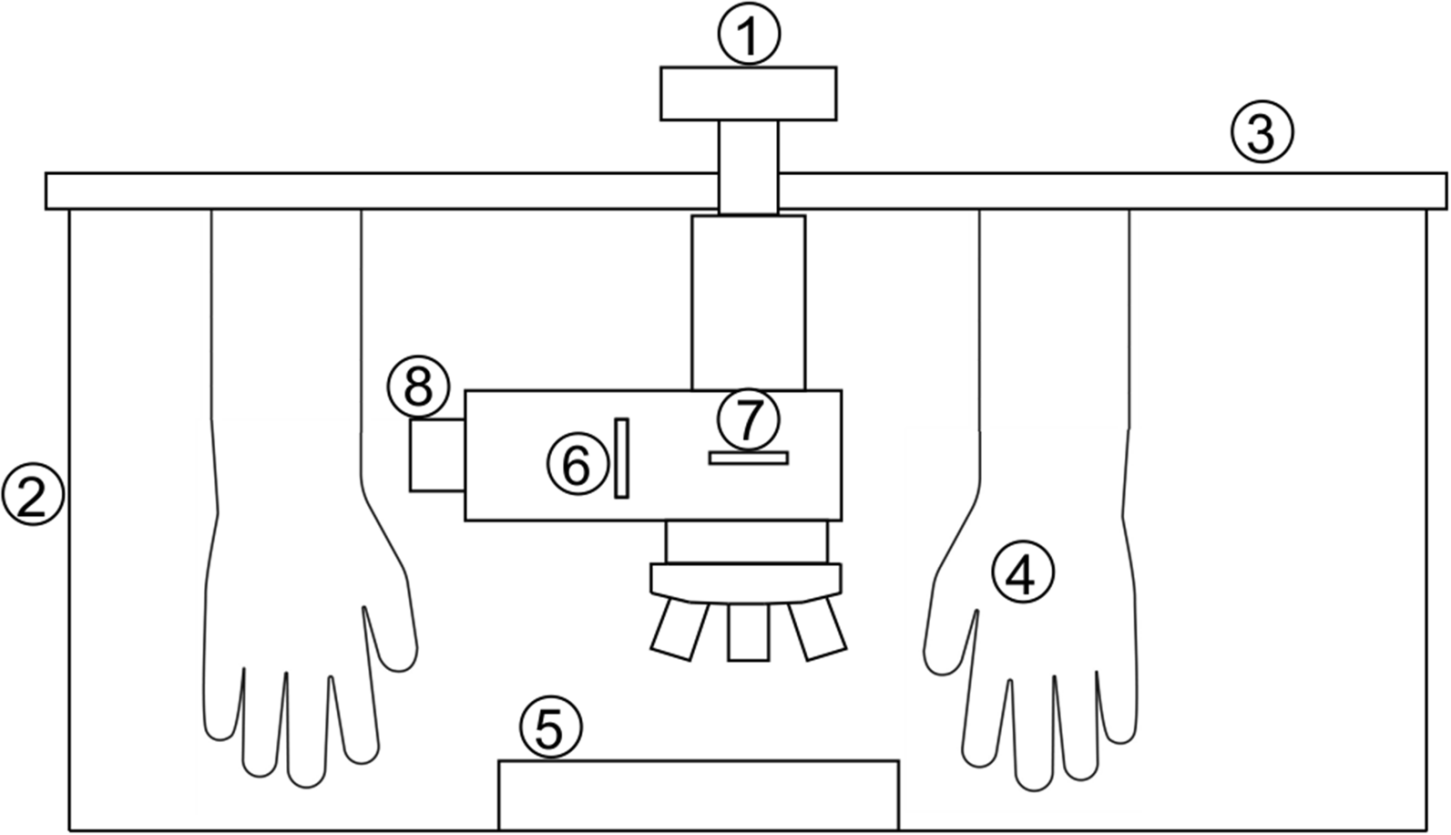
Experimental setup for CM_rpl_: The microscope, excluding the camera unit (1), was placed inside the fully temperature-controlled environment of a laboratory chest freezer (2). A customised transparent acrylic glass lid (3) substituted the standard lid. The lid was equipped with insulated gloves (4) for manipulation inside the temperature-controlled environment. For the microscopic investigations, leaves were clamped into the sample holder (5) or buds with approx. 1 cm long shoots placed on top of it. Orientation of the polarised light was controlled by the polariser (6) and the analyser (7). Illumination was provided by an external cold light source, which was connected to the microscope with fiber optics (8)

For the freezing experiment, whole *Buxus* and *Galanthus* leaves or shoots of *Picea* were cooled down to the respective species specific sublethal target temperatures. To avoid artificial supercooling, cooling rates were limited to a maximum of 3 K/h [39] and a few droplets of a suspension with INA (ice nucleation active) bacteria (*Pseudomonas syringae* van Hall 1902) were added into the Eppendorf tubes. Upon reaching the target temperatures, leaves and buds plus shoots were sectioned in the frozen state inside the cooling compartment with a similarly cooled razorblade by use of insulated gloves that are build-in in the lid of the freezer (Fig. 4). Apart from this, cutting was performed in the same way as described for control samples above. Immediately afterwards (< 30 s) the cut surfaces were inspected and imaged.

### Microscopic system

The experiments were performed with a reflected-light microscopic system under temperature-controlled conditions. All manipulation steps were exclusively executed in a temperature-controlled environment to prevent condensation effects (Fig. 4).

A customisable BXFM-F microscope unit containing the following components (all parts from Olympus, Tokyo, Japan) was used: Reflected-light illuminator U-KMAS mounted on BXFM-ILHS, single port tube with lens U-TLU and a video camera mount adapter U-CMAD3. Illumination was provided by an external cold light source (KL1600LED, Schott, Mainz, Germany). For magnification, the microscope was equipped with the following objective lenses: MPLFLN5X (NA = 0.15, WD = 20 mm), MPLFLN10X (NA = 0.3, WD = 11 mm), LMPLFLN20X (NA = 0.4, WD = 12 mm) (Olympus, Tokyo, Japan). Focusing was conducted by a motorised stage M-MFD-MX51 (Märzhäuser Wetzlar, Wetzlar, Germany). Images were taken with a 9 megapixel digital camera (UC90), which can be fully controlled by the image analysis software cellSense Standard (Olympus, Tokyo, Japan). For the investigation of optical properties (e.g. birefringence) a polariser (U-PO3) and a rotatable analyser (U-AN360-3) (both from Olympus, Tokyo, Japan) were used.

### Controlled freezing treatment

The microscopic system except for the camera unit was placed inside of a laboratory chest freezer (ProfiLine Taurus PLTA0987, National Lab, Moelln, Germany) (Fig. 4) which is fully temperature controlled. For thermal insulation the lid of the laboratory chest freezer was substituted by a customised acrylic glass lid. The lid was equipped with thermally insulated gloves, which allowed manipulations inside the cold compartment during the freezing treatment. Control technology enabled to regulate the temperature inside the laboratory chest freezer [54]; hence simulation of temperature profiles with defined cooling/warming rates was possible. Input variables for the desired temperature treatment were set with a control software (programmed in Lab View by O. Buchner). Precise temperature control was experimentally tested between ambient temperature and − 21 °C. However, the cooling capacity of the laboratory chest freezer would be − 86 °C, hence lower target temperatures could be possible, when necessary. Removing the acrylic glass lid from the cooled laboratory chest freezer upon completion of a freezing experiment caused intense condensation on the microscope; this was eliminated by rewarming without removing the lid and increasing the temperature with multiple heating pads (total heating power = 49.8 W) attached to the microscopic unit. Controlled rewarming ensured that the temperature of the microscope was always higher than the air inside of the cooling compartment, which completely ruled out condensation effects.

## Data Availability

The datasets used and/or analysed during the current study are available from the corresponding author on reasonable request.

## Abbreviations

CM_rpl_: Cryo-microscopy in reflected-polarised-light
Cryo-SEM: Cryo-scanning electron microscopy
DTA: Differential thermal analysis
IDTA: Infrared differential thermal analysis
MRI: Magnetic resonance imaging
NMR: Nuclear magnetic resonance
INA: Ice nucleation active bacteria
